# Morphological Changes Induced by TKS4 Deficiency Can Be Reversed by EZH2 Inhibition in Colorectal Carcinoma Cells

**DOI:** 10.3390/biom14040445

**Published:** 2024-04-05

**Authors:** Mevan Jacksi, Eva Schad, Agnes Tantos

**Affiliations:** 1 HUN-REN Research Centre for Natural Sciences, 1117 Budapest, Hungary; mehvan.jacksi@uoz.edu.krd (M.J.); schad.eva@ttk.hu (E.S.); 2 Doctoral School of Biology, Institute of Biology, ELTE Eötvös Loránd University, 1053 Budapest, Hungary; 3 Department of Biology, College of Science, University of Zakho, Duhok 42002, Iraq

**Keywords:** EZH2, enhancer of zeste homolog 2, TKS4, signal transduction, long non-coding RNA, migration, invasion, EMT, epithelial–mesenchymal transition, Frank–Ter Haar syndrome

## Abstract

Background: The scaffold protein tyrosine kinase substrate 4 (TKS4) undergoes tyrosine phosphorylation by the epidermal growth factor receptor (EGFR) pathway via Src kinase. The TKS4 deficiency in humans is responsible for the manifestation of a genetic disorder known as Frank–Ter Haar syndrome (FTHS). Based on our earlier investigation, the absence of TKS4 triggers migration, invasion, and epithelial–mesenchymal transition (EMT)-like phenomena while concurrently suppressing cell proliferation in HCT116 colorectal carcinoma cells. This indicates that TKS4 may play a unique role in the progression of cancer. In this study, we demonstrated that the enhancer of zeste homolog 2 (EZH2) and the histone methyltransferase of polycomb repressive complex 2 (PRC2) are involved in the migration, invasion, and EMT-like changes in TKS4-deficient cells (KO). EZH2 is responsible for the maintenance of the trimethylated lysine 27 on histone H3 (H3K27me3). Methods: We performed transcriptome sequencing, chromatin immunoprecipitation, protein and RNA quantitative studies, cell mobility, invasion, and proliferation studies combined with/without the EZH2 activity inhibitor 3-deazanoplanocine (DZNep). Results: We detected an elevation of global H3K27me3 levels in the TKS4 KO cells, which could be reduced with treatment with DZNep, an EZH2 inhibitor. Inhibition of EZH2 activity reversed the phenotypic effects of the knockout of TKS4, reducing the migration speed and wound healing capacity of the cells as well as decreasing the invasion capacity, while the decrease in cell proliferation became stronger. In addition, inhibition of EZH2 activity also reversed most epithelial and mesenchymal markers. We investigated the wider impact of TKS4 deletion on the gene expression profile of colorectal cancer cells using transcriptome sequencing of wild-type and TKS4 knockout cells, particularly before and after treatment with DZNep. Additionally, we observed changes in the expression of several protein-coding genes and long non-coding RNAs that showed a recovery in expression levels following EZH2 inhibition. Conclusions: Our results indicate that the removal of TKS4 causes a notable disruption in the gene expression pattern, leading to the disruption of several signal transduction pathways. Inhibiting the activity of EZH2 can restore most of these transcriptomics and phenotypic effects in colorectal carcinoma cells.

## 1. Introduction

Scaffold proteins govern cellular signaling by engaging in interactions and bringing various pathway components into close proximity, including enzymes and regulatory proteins [1]. The scaffold protein TKS4, encoded by the *SH3PXD2B* gene, is a member of the p47 organizer protein family that contains four Src homology 3 domains (SH3) and a phox homology domain (PXD) [2]. TKS4 is associated with Src substrate adaptor proteins, which are essential for the production of podosomes and the invasion and growth of cancer cells [2,3]. TKS4 deletion or mutation in humans results in the appearance of a hereditary disorder known as FTHS [4,5,6], which is characterized by abnormal growth, skeletal abnormalities, and changes in facial features such as a large cornea, a flattened back of the head, a large cheek, and a small chin [7]. Similar phenotypic changes could be induced by the knockout of TKS4 in mice in an FTHS model. The TKS4-deficient mice show aberrant development resulting in stunted growth, craniofacial and skeletal deformities, glaucoma, hearing impairment, and a virtual lack of white adipose tissue [8]. TKS4 can regulate EMT-like processes through a mechanism not yet clearly defined [9,10]. Modifications in the differentiation of cell lineages and the maturation of cells were observed upon TKS4 depletion that can be connected to the development of FTHS [11]. The protein is mostly situated in the cytoplasm of inactive cells, but it relocates and binds to the membrane when exposed to epidermal growth factor (EGF) stimulation. This relocation is likely mediated by the Src kinase, which acts as a connector between TKS4 and EGFR [5]. EGFR is autophosphorylated upon EGF stimulation, which causes Src tyrosine phosphorylation. Then, TKS4 binds to the SH3 domain of Src through its proline-rich motif. The kinase domain of Src phosphorylates tyrosine 25 of TKS4’s PX domain, tyrosine 373 of its 3rd SH3 domain, and tyrosine 508 of its disordered region. The extended interaction between TKS4 and Src enhances the stability of the kinase and enables further substrate phosphorylation [12]. In summary, existing evidence indicates that TKS4 plays a central organizing role in several signal transduction pathways, and its presence is necessary for proper development and cellular function. However, the details of the molecular mechanisms of the protein’s diverse effects are not fully understood yet.

The Polycomb Group (PcG) proteins, particularly PRC1 and PRC2, are widely recognized as mediating developmental gene silencing [7,13]. PRC1 and PRC2 have the ability to control the genomic structure and repress gene expression during the developmental process [14]. Genomic domains are classified into two categories: active domains and repressive domains. Both types regulate gene expression and determine the specific characteristics of a cell. The active domains comprise the genes involved in cell self-renewal, which are guided by super-enhancers, whereas the repressed domains include the genes marking the repressed lineage [15]. Furthermore, it has been shown that intact PcG domains are necessary for preserving the chromatin interaction landscape. Nevertheless, the precise mechanisms governing the formation of PcG domains and the complete understanding of PcG protein recruitment remain elusive, making the investigation of silencers more challenging [16,17,18,19,20].

Enhancer of zeste homolog 2 (EZH2) is a histone lysine methyltransferase responsible for the activity of PRC2, maintaining the trimethylated lysine 27 mark on histone H3 (H3K27me3) [21]. H3K27 trimethylation can be catalyzed to a lesser extent by the enhancer of zeste homolog 1 (EZH1), too [22]. H3K27me3 marks are associated with the regulation of genes specific to particular cell types. H3K27me3 is a known feature of silencers [17,23,24]. As a central factor in gene expression regulation, EZH2 is well-known for its involvement in EMT [25,26,27] and in several different signaling pathways [28,29]. Overactivation is observed in several cancers [30,31], and higher EZH2 activity is generally accompanied by a worse disease prognosis and a more aggressive phenotype. Because of its recognized role in cancer progression, regulating EZH2 activity is considered an important anti-tumor therapeutic method [32,33,34]. Since TKS4 is not directly involved in gene expression regulation, the previous observations regarding the induction of EMT-like processes [9] and the dysregulation of a wide variety of signaling pathways upon the deletion of TKS4 [35] raise the possibility of an indirect mediator that conveys the removal of TKS4 into altered gene expression patterns. Based on the overlaps between the roles of EZH2 and the observed changes in the TKS4 KO cells, we hypothesized that this mediator may be EZH2 itself. Therefore, in this work, we investigated the role of EZH2 in the cellular effects of TKS4 deletion in colorectal carcinoma cells with the aim of better understanding the molecular networks relying on TKS4.

## 2. Materials and Methods

### 2.1. Cell Culture and TKS4 Knockout

The TKS4 (gene ID: 285590) KO HCT116 cell line was created in accordance with the instructions provided in a 2019 publication [9]. HCT116 cells were grown in McCoy’s 5A medium (Life Technologies, Paisley, UK) supplemented with 10% fetal bovine serum (FBS, Life Technologies, Paisley, UK) and 1% penicillin/streptomycin (Life Technologies, Paisley, UK). All cells were grown at 37 °C in a humidified atmosphere with 5% CO_2_ [36]. The TC20 Automated Cell Counter (Bio-Rad, Hercules, CA, USA) was used to measure the number and viability of cells using 0.4 percent trypan blue dye exclusion [37]. An examination of the morphology was conducted utilizing an Axiovert 25 inverted microscope (Carl Zeiss Microscopy, Jena, Germany). Cell line authentication performed by Eurofins Genomics Europe Shared Services GmbH (Eurofins, Ebersberg, Germany) confirmed the 100% authenticity of the cells.

### 2.2. EZH2 Inhibitor Treatment

**Figure 1:**
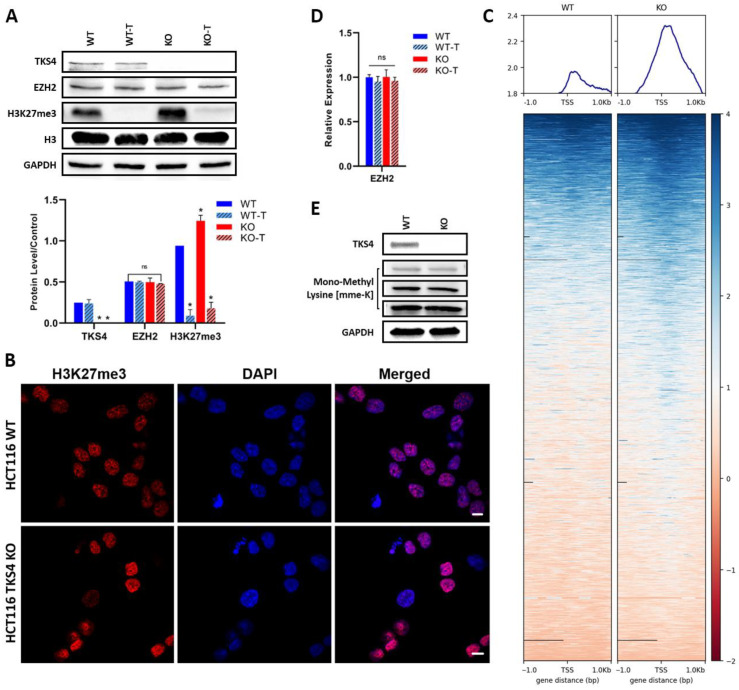
The effect of TKS4 deletion on H3K27me3. (**A**) Top: Western blot images of TKS4 and EZH2 H3K27me3. Histone 3 (H3) and GAPDH were used as protein loading controls. Bottom: analysis of Western blotting images (*n* = 3). (**B**) Immunocytochemistry images show the H3K27me3 levels in WT and TKS4 KO HCT116 cells. Nuclei were stained with DAPI. (**C**) Heatmap visualization of the H3K27me3 ChIP-seq data (RPGC normalized). Heatmaps are sorted by ChIP-seq signal in descending order. EZH2 RNA expression levels (**D**) and global monomethylation levels (**E**) in the different cell lines and treatments. Scale bar: 20 µm unmodified (WT), EZH2 activity inhibitor-treated (WT-T), TKS4-deficient (KO), and TKS4-deficient treated with EZH2 activity inhibitor (KO-T) HCT116 cells. Results significant at *p* > 0.005 are marked with an asterisk, ns: non-significant. Original Western blot images are presented in Supplementary Figure S1A.

3-deazaneplanocin A (DZNep) (Chem-Cruz, sc-351856) is an effective S-adenosylhomocysteine hydrolase inhibitor, which is a small-molecule inhibitor of EZH2 activity [38]. A cell viability assay and Western blotting were conducted to determine the optimal concentration of DZNep for its impact on cell viability and determine the proper concentration (Supplementary Figure S1C). A concentration of 1 µM of DZNep had enough EZH2 inhibition activity (Figure 1A) to lower dead cells; therefore, 1 µM was selected for the downstream experiments. HCT116 cells were cultured untreated or treated with DZNep for 48 h in a 37 °C, 5% CO_2_ humidified incubator before being harvested for downstream investigations.

### 2.3. Total RNA Extraction and Real-Time Quantitative PCR

To lyse and isolate the total RNA of HCT116 cell lines, TRIzol^TM^ Reagent (Life Technologies, Carlsbad, CA, USA) and the Direct-zol^®^ MiniPrep kit (Zymo Research, Irvine, CA, USA) were used. DNA contamination is eliminated following a 15 min DNAse I (Zymo Research, Irvine, CA, USA) treatment. The quality and quantity of the RNAs were determined using the nanophotometer IMPLEN (IMPLEN, München, Germany). Complementary DNA (cDNA) was synthesized from 1 µg of total RNA using the First Strand cDNA Synthesis Kit for RT-PCR (AMV) (Roche, Basel, Switzerland) and SuperScript™ III Reverse Transcriptase (Life Technologies, Paisley, UK). The real-time PCR experiments were carried out using TaqMan™ Fast Advanced Master Mix (Life Technologies, Paisley, UK) by QuantStudio™ 5 and 6 Pro Real-Time PCR System (Life Technologies, Paisley, UK). To determine the relative expression, three replicates were used based on a 2^−ΔΔCt^ method for fold change determination [39]. Probes of TaqMan^®^ assays provided by Thermo Fisher Scientific are listed in Supplementary Table S4B.

### 2.4. Whole Transcriptome Sequencing (RNA-seq)

The total RNA of different conditions of HCT116 cells and their quality and quantity were isolated using the previously mentioned RNA extraction section. A total of 1 µg of RNA from each sample was used after rRNA depletion on an Illumina NovaSeq 6000 high-throughput next-generation sequencing (NGS) platform (Novogene, Cambridge, UK) for whole-transcriptome sequencing [40]. Three biological replicates for the WT and KO were prepared, and two replicates for the samples treated with EZH2 activity inhibition were prepared.

### 2.5. RNA Sequencing Data Analysis

The acquired 150 bp paired-end reads were aligned to the GRCh38 human transcriptome by using and applying HISAT2, according to Kim et al. 2019 [41]. Aligned reads are converted to BAM format, followed by sorting and indexing using the samtools [42]. The Cufflinks program package (cufflinks, cuffmerge, cuffquant, and cuffdiff) [43] was utilized to conduct transcriptome assembly and differential expression analysis. Changes in expression levels were computed by comparing the FPKM (fragments per kilobase of exon per million mapped fragments) values of the wild-type and knockout samples. Variations are represented as base two logarithms (log2 fold change) of the ratio between the knockout and the wild-type samples. Heatmaps and volcano plots were generated by the CummeRbund R package [44] and GraphPad Prism 8.0.2 software (GraphPad Software, San Diego, CA, USA), respectively.

### 2.6. Protein Extraction and Western Blot Analysis

HCT116 cells were washed twice with cold 1X PBS, and total proteins were extracted using an appropriate amount of RIPA buffer (1% NP-40, 1% SDS, 1% sodium deoxycholate, 150 mM NaCl, 25 mM Tris-HCl pH 7.6) with the addition of a protease inhibitor cocktail (10 μL/mL) (Life Technologies, Paisley, UK). Using a prechilled centrifuge, the cell debris was pelleted at 18,000× *g* for 15 min. Protein concentration determination was carried out using the Pierce™ BCA Protein Assay Kit (Life Technologies, Paisley, UK). Protein amounts of 25 to 30 µg were separated using a 4–20% gradient TGX gel (BioRad, Hercules, CA, USA) and followed by transfer to nitrocellulose and PVDF membranes (BioRad, Hercules, CA, USA). The membrane was blocked with 5% non-fat milk in Tris-buffered saline containing 0.05% Tween-20 (TBS-T) for one hour at room temperature; consequently, the membrane was washed three times with TBS-T and incubated overnight at 4 °C with different primary antibodies. Corresponding secondary antibodies (Supplementary Table S4A) were used and incubated for 1 h at room temperature after three washing steps with TBS-T. Enhanced chemiluminescence (ECL) reagent (BioRad, Hercules, CA, USA) was used to develop the membranes, and the signals generated were captured using the ChemiDoc^TM^ imaging system (BioRad, Hercules, CA, USA). The ImageJ (version 1.53) and ImageLab (version 6.1) software were applied to perform densitometry of the Western blot bands [45].

### 2.7. Immunocytochemistry and Fluorescence Microscopy

Cells were seeded at a density of 35,000 cells per well onto a rounded cover slip (VWR, Leicestershire, UK). Once confluence had reached 80%, the media was drained, and three washes were carried out with 500 L of pre-warmed 1X PBS on the cells. The cells were fixed for 15 min at room temperature in a 4% paraformaldehyde solution. Following a second round of washing, the cells were blocked at room temperature for one hour using 500 µL of complete blocking solution (0.2% gelatin, 2% BSA, 5% FBS, and 0.1% Triton X-100, completed with 1X PBS). Subsequently, the cells were incubated at 4 °C overnight with the corresponding appropriate primary antibodies, as specified in Supplementary Table S4A. Following incubation, the cells were incubated with the corresponding secondary antibodies for one hour at room temperature after being rinsed three times with 1X PBS. DAPI was applied to stain nuclei (Merck KGaA, Darmstadt, Germany). A confocal microscopy system (ZEISS LSM-710 camera) was used to capture the images (Carl Zeiss Microscopy GmbH, Jena, Germany). ZEN 3.2 software was utilized for image processing (Carl Zeiss Microscopy GmbH, Jena, Germany).

### 2.8. Chromatin Immunoprecipitation Sequencing (ChIP-Seq)

ChIP-seq was performed according to the manufacturer’s protocol using the Pierce™ Magnetic ChIP Kit (Life Technologies, Paisley, UK). Cells were sonicated at 18 cycles, 30 s ON/30 s OFF (high setting), using the Bioruptor (Diagenode, Seraing, Belgium). The cell lysates were incubated overnight at 4 °C with the H3K27me3 monoclonal antibody (G.299.10), ChIP-verified (Life Technologies, Paisley, UK), and rabbit IgG (Sigma, St. Louis, MO, USA) negative control antibody provided by the kit. A total of 1 µg of DNA from each sample was sent for the construction of a cDNA library for Illumina high-throughput NGS sequencing (Novogene, Cambridge, UK).

### 2.9. ChIP-Seq Data Analysis

As a first step, we performed quality control with FastQC (version 0.12.0), available at: http://www.bioinformatics.babraham.ac.uk/projects/fastqc/. The KO sample contained a considerable amount of adapter sequences, so Trimmomatic, a read trimming tool [46], was used to eliminate them. The sequenced reads (in Fastq format) were mapped to the human reference genome GRCh38 (version GRCh38.d1.v1) using BWA-MEM (version 0.7.1) [47]. The aligned SAM files were converted to BAM files using SAMtools View (version 1.10) [42], and the BAM files were sorted with SAMtools sort and indexed with SAMtools index. Duplicates (reads located at the exact same location) were marked using Picard tools MarkDuplicates (version 1.100) [http://broadinstitute.github.io/picard/]. For visualization of ChIP-seq data, coverage files were created that show read numbers per genomic region using deepTools bamCoverage (version 3.1.3.) [48]. The RPGC (Reads Per Genomic Content) normalization method was applied. In order to obtain information about the methylation in the promoter regions, read density plots and heatmaps were created around TSS. To achieve that, we first created an intermediate matrix file using deepTools computeMatrix. Heatmaps combined with read density profiles were created using deepTools plot Heatmap.

### 2.10. Cell Migration Assay 

The cell migration assay [49] was carried out using µ-Dish culture-insert 2 wells (Ibidi GMBH, Gräfelfing, Germany). Following the manufacturer’s instructions, 7 × 10^4^ cells were placed in culture-insert dishes and allowed to incubate until they attained full confluency. Subsequently, the plastic inserts were taken out, and the dishes were rinsed using PBS and resupplied with 1 mL of McCoy’s 5A complete medium (Life Technologies, Paisley, UK) before being placed in an incubator at a temperature of 37 °C to facilitate the wound closure. The LEICA DMi1 inverted microscope (Leica Microsystems GmbH, Wetzlar, Germany) was used to capture images at 0 h, 24 h, and 48 h.

### 2.11. Transwell Invasion Assay

A cell invasion assay [49] was carried out for HCT116 wild-type and knockout using BioCoat^®^ Matrigel^®^ Invasion Chambers (Corning Inc., Corning, NY, USA). In the upper chambers, cells were seeded at a density of 2.5 × 10^4^, filled with McCoy’s 5A serum-free medium, and incubated for 48 h. As a chemoattractant, 5% FBS was added to the lower chamber. Using a cotton-tipped swab, the cells on the inserts’ upper surface were removed after 20, 40, and 60 h of incubation time, and the cells presented on the bottom surface of the insert were fixed with 100% methanol, followed by staining with crystal violet. Images were captured with a Leica DMi1 inverted microscope. Invasion efficiency was determined by invading cells counted in 10 random fields at 10X objective lens magnifications and analyzed.

### 2.12. Cell Proliferation Assay

In a 24-well plate, 1 × 105 cells were seeded, each containing 1 mL of McCoy’s 5A complete media. Following adhesion, cells were washed twice with 1X PBS, fixed with a 10% formalin solution, and marked as day 0. From days 2 to 5, cells were fixed daily. Cells were washed three more times with PBS and then left to dry. A total of 500 µL of 0.5 crystal violet (Merck, Darmstadt, Germany) was used to stain the cells. Crystals were resolubilized by adding 10% acetic acid (Merck, Darmstadt, Germany) to each well. At 595 nm, the absorbance was read using a SynergyMX plate reader (BioTek Instruments, Winooski, VT, USA).

### 2.13. Viability Assay

The viability of cells was determined by adding equal volumes of 0.4% trypan blue dye to the cells suspended in serum-free McCoy’s 5A medium, followed by an incubation of three minutes at room temperature. A hemocytometer counter was used to count the cells using a Leica DMi1 inverted microscope (Leica Microsystems GmbH, Wetzlar, Germany). The percentage of viable cells was determined by calculating the number of viable cells divided by the number of total cells. 

### 2.14. Statistical Analysis

An unpaired Student’s *t*-test was performed to analyze the statistics of the experimental data, and GraphPad Prism 8.0.2 software was used (GraphPad Software, San Diego, CA, USA).

## 3. Results

### 3.1. Elevation of H3K27me3 in TKS4 KO Cells

In our previous work [35], we demonstrated that the removal of TKS4 results in a fundamental reorganization of several different signaling pathways, which was accompanied by large-scale changes in the transcriptome of the TKS4 KO human colorectal cancer (HCT116) cells. In our pursuit to understand the molecular mechanisms behind these changes, our attention was drawn to EZH2, an important regulator of gene expression whose effects are similar to the phenotypic changes observed in the KO cells, and the Cancer Genome Atlas (TCGA) database revealed that the EZH2 expression was 2.4 times higher in colon cancer compared to normal cells [50]. Therefore, we compared the activity of EZH2 in the WT HCT116 and the TKS4 KO cells using Western blot, immunocytochemistry (ICC), and chromatin immunoprecipitation (ChIP-seq) against the H3K27me3 mark. Our results, represented in Figure 1A, showed that the global H3K27me3 level was elevated in the TKS4 KO cells, with an approximately 1.3-fold increase compared to the WT cells. An increased H3K27me3 intensity could also be observed by ICC (Figure 1B), where the H3K27me3 labeling resulted in stronger signals in the KO cells than in the WT. While the 1.3-fold increase is not a dramatic change, it can still lead to altered gene expression, and its effects can be propagated throughout the proteome if key regulators are affected. Our ChIP-seq results confirmed these alterations, as we could detect a similar elevation in the H3K27me3 mark in the promoter region of genes (Figure 1C and Figure S1B). Importantly, these changes were not a result of an increased availability of EZH2, as the RNA expression and protein level of EZH2 appeared to be unchanged, indicating that the higher methylation level was due to its increased activity (Figure 1A,D). The observed changes in H3K27me3 levels were not accompanied by a general increase in protein methylation, as a Western blot with mono-methyl lysine antibody confirmed that the monomethylated lysine levels were unchanged in the KO cells compared to the WT (Figure 1E).

### 3.2. The Absence of TKS4 Induces Migration and EMT-Like Changes through EZH2 Hyperactivity

**Figure 2:**
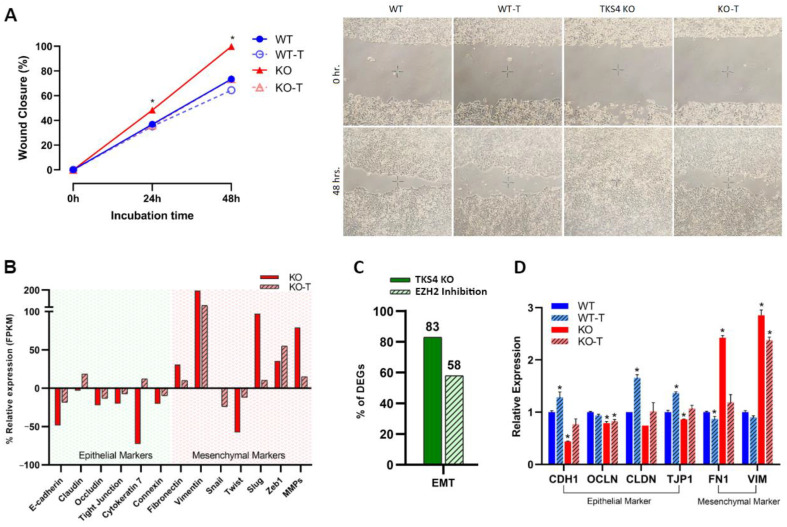
Effects of TKS4 and EZH2 inhibition on cell motility. (**A**) Left: migration speed (*n* = 3) of the WT (blue), WT-T (dotted line, empty circles), KO (red), and KO-T (dotted line, empty triangles) cells. Right: representative wound healing assay images show cell migration of HCT116 cells in 0 h after plastic inserts were removed (top row) and 48 h after plastic inserts were removed (bottom row). (**B**) Relative expression of epithelial and mesenchymal markers based on transcriptome sequencing data. The expression of the genes in the untreated WT cells was set to 0. (**C**) Percentage of differentially expressed genes (DEGs) in the KO and the KO-T cells. (**D**) Expression levels of epithelial and mesenchymal markers based on qPCR experiments (*n* = 3). Results significant at *p* > 0.005 are marked with an asterisk.

The deletion of TKS4 generated numerous morphological alterations in the HCT116 cells, in keeping with the previously documented EMT-like characteristics [9,35]. To determine if there is a causal relationship between the observed morphological changes and the activation of EZH2, we applied an EZH2 activity inhibitor treatment. A total of 1 µM DZNep treatment resulted in a significant reduction of H3K27me3 levels in both the WT and the KO cells (Figure 1A), indicating the efficiency of the treatment. DZNep is an inhibitor of EZH2 activity; therefore, it allowed us to directly investigate the effects of EZH2 inhibition itself rather than the disruption of the PRC2 complex. EZH2 activity inhibition resulted in a reversal of the increased migratory speed of the KO cells, while it was much less effective in the WT cells. Compared to the WT cells, the TKS4 KO cells demonstrated a 30% increase in wound closure after 48 h, while this difference was reduced to 10% between the KO and WT DZNep-treated cells (Figure 2A and Figure S2A). Even more importantly, the inhibitor-treated KO cells showed similar behavior to the WT cells, indicating that EZH2 inhibition could counter the effects of TKS4 deletion. The fact that there was no significant difference in the behavior of the non-treated and treated WT cells suggests that the observed effects are linked to the absence of TKS4 and not an inherent consequence of EZH2 inhibition. 

The increased motility of the TKS4 KO cells was accompanied by a downregulation of epithelial markers such as e-cadherin (CDH1) [51], claudin (CLDN) [52], occluding (OCLN) [53], tight junction protein (TJP1) [53,54], cytokeratin (KRT7) [55], and connexin (CNST) [56,57] and an increase in the expression level of most mesenchymal markers, such as fibronectin-1 (FN1) [58], vimentin (VIM) [59], snail (SNAI1) [60], slug (SNAI2) [61], twist (TWIST) [62], matrix metalloproteinases (MMPs) and ZEBs (ZEB1) [63] (Figure 2B and Supplementary Figure S2B). On a whole-transcriptome level, RNA-seq data indicated that about 80% of well-known EMT markers are changed upon deletion of TKS4. These changes are mostly restored when EZH2 activity is inhibited in the KO cells (Figure 2C and Supplementary Tables S1A and S2). Treating the WT cells with an EZH2 inhibitor generally increases the expression of epithelial markers and decreases the mesenchymal markers, as shown in the EMT section of Supplementary Table S1.

Targeted qPCR measurements confirmed the findings of the transcriptomic analysis (Figure 2D): epithelial markers generally showed a reduced expression in the KO cells, mesenchymal markers were dramatically increased, and EZH2 inhibitor treatment could largely reverse these alterations. In the case of the WT cells, inhibitor treatment resulted in a milder but similar outcome, indicating that the EMT-like changes observed in the TKS4 KO cells are directly related to the activity of EZH2.

### 3.3. Absence of TKS4 Induces Invasion through EZH2 Hyperactivity

**Figure 3:**
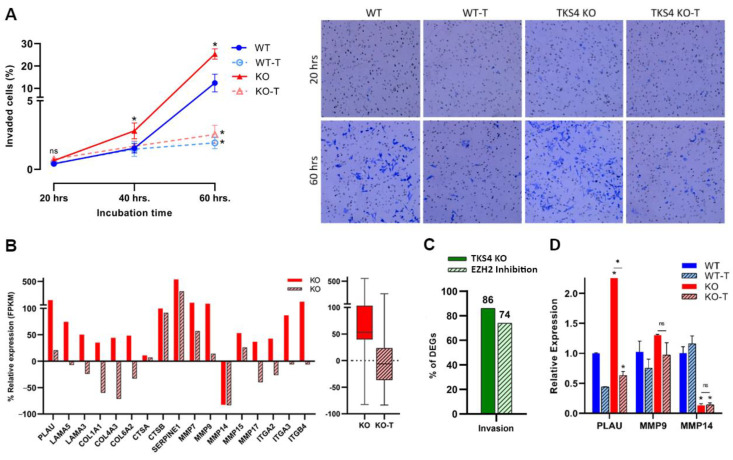
Effects of TKS4 and EZH2 inhibition on the invasion capacity. (**A**) Left: invasion speed of the WT (blue), WT-T (dotted line, empty circles), KO (red), and KO-T (dotted line, empty triangles) cells. Right: representative invasion assay images show the number of invaded cells in 20 h after cell seeding (0 h after the cells on the upper surface of the inserts were removed) (top row) and 60 h after cell seeding (bottom row). Original invasion assay images are presented in Supplementary Figure S3. (**B**) Left: relative expression of individual invasion markers based on transcriptome sequencing. Right: relative expression (FPKM) of total invasion markers based on transcriptome sequencing. (**C**) Percentage of DEGs in the KO and the KO-T cells. (**D**) Expression levels of invasion markers based on qPCR experiments (*n* = 2). Results significant at *p* > 0.005 are marked with an asterisk, ns: non-significant.

The absence of TKS4 not only increased the speed of migration but also enhanced the capability for invasion, with the number of invading cells doubling by 60 h in the case of TKS4 KO cells, compared to the WT. In line with the known involvement of EZH2 in the invasion of cancer cells [64], EZH2 inhibition significantly reduced the invasion capacity of both the WT and the KO cells (Figure 3A and Supplementary Figure S3A).

According to the transcriptomic analysis, a noticeable elevation could be observed in the expression of most of the invasion marker genes (Figure 3B and Figure S3B), such as urokinase-type plasminogen activator (PLAU) [65], laminin genes (LAMA3, LAMA5) [66,67,68], collagen-related genes (COL1A1, COL4A3, and COL6A2) [69,70,71,72], cathepsin genes (CTSA, CTSB) [73,74,75], serpine1 (PAI-1) [76], MMP genes (MMP7, 9, 13, 14, and 17) [77,78,79,80,81], and integrin-related genes (ITGA2, ITGA3, and ITGB4) [82,83,84]. The analysis also revealed that over 85% of recognized invasion markers undergo alterations upon TKS4 deletion, and around 75% of these changes are reversed by the inhibition of EZH2 activity (Figure 3C and Supplementary Tables S1A and S2). Quantitative measurements confirmed an increase in the expression level of an invasion marker, urokinase-type plasminogen activator (PLAU) (increase above 2.5-fold), and MMP9, which decreased significantly upon EZH2 inhibitor treatment. On the other hand, they also indicated a strong downregulation of MMP14 expression in TKS4 KO cells that could not be changed with the inhibitor treatment (Figure 3D). Immunostaining showed that the localization of MMP14 is also perturbed by TKS4 deletion, as the protein was more precisely located around the nucleus of the TKS4 KO cells, with a decreased protein level compared to MMP14 observed in the spindles (invadopodia and podosome) of WT cells (Supplementary Figure S8).

### 3.4. EZH2 Inhibition Decreases Proliferation Rate

**Figure 4:**
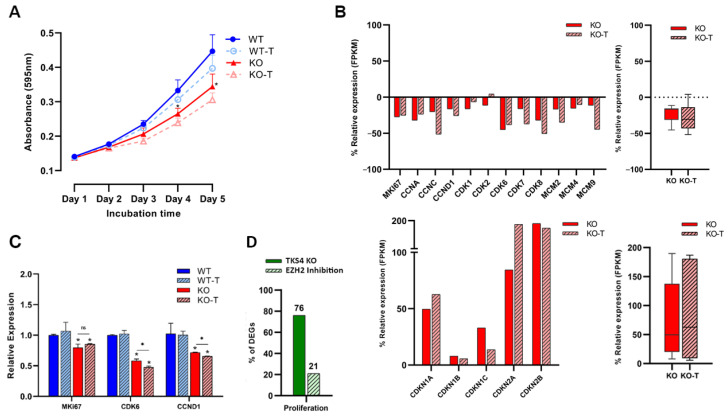
Effects of TKS4 and EZH2 inhibition on proliferation. (**A**) Proliferation rates of the WT (blue), WT-T (dotted line, empty circles), KO (red), and KO-T (dotted line, empty triangles) cells (*n* = 3). (**B**) Top left: relative expression of the individual proliferation promoter genes based on transcriptome sequencing. Top right: relative expression of total proliferation promoters based on transcriptome sequencing. Bottom left: relative expression of individual proliferation inhibitors based on transcriptome sequencing. Bottom right: relative expression (FPKM) of total proliferation inhibitors based on transcriptome sequencing. (**C**) Expression levels of selected proliferation markers based on qPCR experiments (*n* = 2). (**D**) Percentage of DEGs in the KO and the KO-T cells. Results significant at *p* > 0.005 are marked with an asterisk, ns: non-significant.

Surprisingly, the cells lacking TKS4 exhibited a notable decrease in cell proliferation rate while simultaneously experiencing an increase in cell motility and invasion potential. During a five-day proliferation assay, both wild-type (WT) and knockout (KO) cells exhibited comparable rates of proliferation until the second day following adhesion. However, the proliferation of the KO cells significantly decelerated starting on the third day, resulting in a drop of about 40% by the fifth day. Contrary to other morphological changes, the EZH2 treatment could not reverse these effects, as the proliferation rate was further reduced after treatment of both wild-type and TKS4 KO cells (Figure 4A). Transcriptomic data demonstrated that the TKS4 KO cells exhibited a decrease in the expression of key proliferation promoter genes, such as Marker of proliferation Ki-67 (MKI67) [85], cyclin-dependent kinase genes (CDK1, CDK2, CDK6, CDK7, and CDK8) [86,87,88,89,90], Cyclin A (CCNA) [91], Cyclin C (CCNC) [92], Cyclin D1 (CCND1) [93], and minichromosome maintenance complex components (MCM2, MCM4, and MCM9), and EZH2 inhibitor treatment resulted in further downregulation (Figure 4B). At the same time, proliferation inhibitors, such as cyclin-dependent kinase inhibitors (CDKN1A, CDKN1B, CDKN1C, CDKN2A, and CDKN2B) [94,95], showed increased expression, which was even more pronounced when EZH2 activity was inhibited (Figure 4B and Figure S4). The gene expression levels of MKI67 [96] exhibited a reduction of around 20% in TKS4 knockout cells; however, this decrease remained statistically unchanged following the EZH2 inhibitor treatment. The expression levels of CCND1 [97] and CDK6 [98] exhibited a reduction of around 25% and 40%, respectively, which were further reduced when EZH2 activity was inhibited (Figure 4D). On a global level, around 75% of the widely recognized proliferation markers face changes when TKS4 is deleted, and only roughly 20% of these are restored by EZH2 inhibition (Figure 4C, Supplementary Tables S1A and S2).

### 3.5. Effects of TKS4 and EZH2 Inhibition on Global Gene Expression

**Figure 5:**
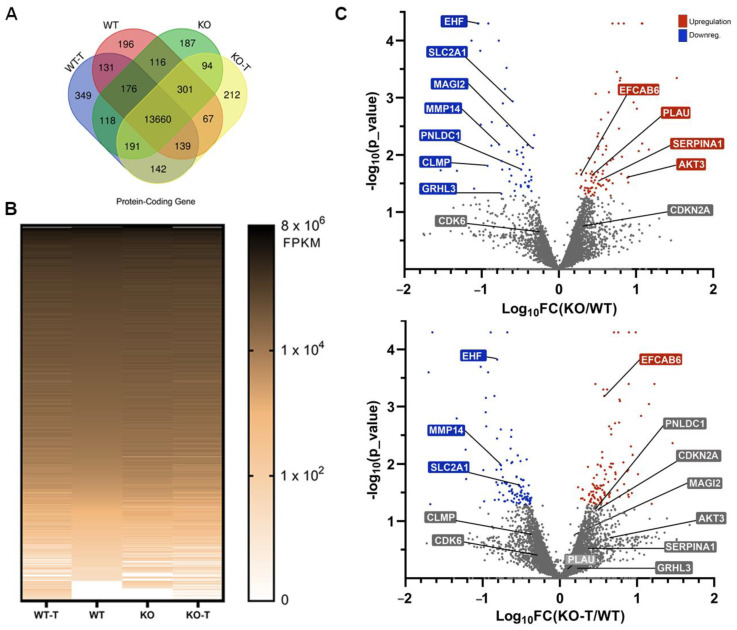
Transcriptome-level changes in mRNAs. (**A**) The Venn diagram of the identified mRNAs in the different cell lines and treatments. (**B**) Heatmap visualization of the DEGs in the different cell lines and treatments. (**C**) Volcano plot of the mRNA expression changes in the KO (top) and the KO-T cells (bottom), with colored points representing significantly upregulated (red) or downregulated (blue) regulated genes (log_10_ *p*-value > 1.3), Insignificance is represented by the color gray.

Approximately 6000 protein-coding genes exhibited a minimum of 25% gene expression alteration following the removal of TKS4, of which 3789 genes were upregulated and 2463 were downregulated, implying a substantial reorganization of the regulation of gene expression following TKS4 deletion. On top of these changes, there are several genes that are only expressed in a particular cell type: 196 and 187 genes were only expressed in WT and KO cells, respectively, while 349 genes were specific to WT-T and 212 genes were specific to KO-T cells (Figure 5A). We also found 142 mRNAs exclusive to the treated cells, suggesting that these genes are strictly regulated by EZH2. As expected, the EZH2 inhibitor-treated samples were characterized by a wider gene expression pattern compared to the untreated cells (Figure 5B). A total of 841 protein-coding genes were significantly downregulated with a log2 fold decrease greater than 1.3 in the TKS4 KO cells, and 1250 were upregulated with a similar efficiency in response to EZH2 inhibition, compared to the WT cells (for details, see Supplementary Table S2).

Several important regulatory genes were significantly downregulated in the KO cells (MAGI2, PNLDC1, CLMP, and GRHL3) that responded to the EZH2 inhibitor treatment (Figure 5C), many of which are involved in epithelial differentiation [99,100,101,102]. We could detect upregulated genes as well (PLAU, SERPINA1, and AKT3), mainly connected to cancer development and progression and mesenchymal phenotypes, that were also responsive to EZH2 inhibition (Figure 5C). The genes belonging to the first group are most probably under the direct control of PRC2 activity, while PRC2 likely regulates the expression of a transcriptional inhibitor in the case of the second.

The presence of several genes unresponsive to EZH2 inhibition indicates that other regulatory mechanisms are connected to TKS4 besides PRC2. Some downregulated genes (EHF and MMP14), mainly involved in proliferation and cancer development, were not restored upon EZH2 inhibitor treatment, and some that were upregulated in KO samples retained or even increased their high expression after treatment (EFCAB6 and CDKN2A) (Figure 5C).

This latter observation offers an explanation for why the proliferation rate was not increased in the EZH2 inhibitor-treated cells. To confirm these findings, some selected genes were also investigated by quantitative PCR (Supplementary Figure S5). The expression of the glucose transporter SLC2A1 (like other proteins belonging to the same family—see Supplementary Table S2) was significantly reduced in the TKS4 KO cells, suggesting that TKS4 plays a role in the signaling pathways that contribute to cancer progression. HOXB7, which controls proliferation and differentiation, was also downregulated. The fact that neither of these changes could be reversed by the addition of an EZH2 inhibitor highlights the importance of other PRC2-independent regulatory pathways connected to TKS4.

As opposed to SLC2A1 and HOXB7, the expression of AKT3 (RAC-gamma serine/threonine protein kinase) and PLAU increased strongly in the TKS4 KO cells and were reduced when the KO cells were treated with the EZH2 inhibitor (Supplementary Figure S5 and Figure 3D). This could suggest that EZH2 controls the expression of a negative regulator; thus, the changes in its activity manifest in reverse in the expression of AKT3 and PLAU.

### 3.6. Alterations in Signaling Pathways

**Figure 6:**
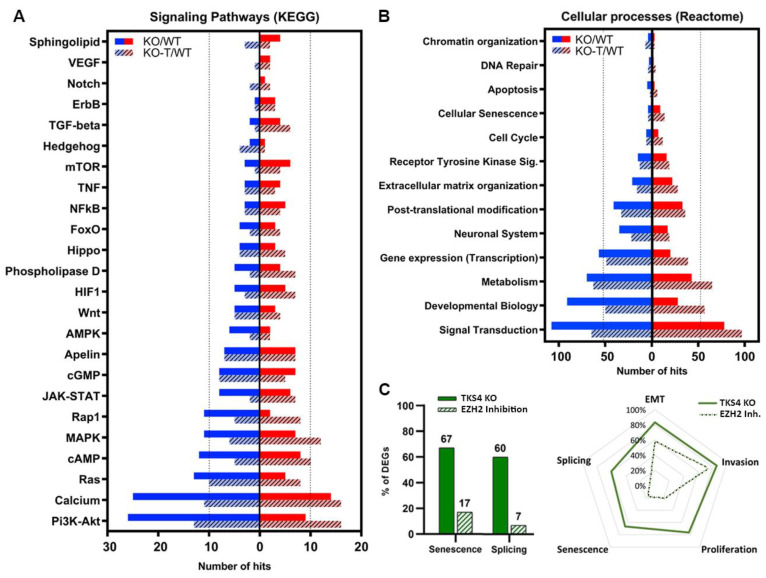
KEGG pathway and Reactome analysis of the transcriptomic changes. (**A**) The effects of TKS4 deletion and EZH2 inhibition on the signaling pathways. (**B**) Effect of TKS4 deletion and EZH2 inhibition on cellular processes. Upregulation is indicated by red, whereas downregulation is indicated by blue. EZH2 inhibitor treatment is indicated by dashed columns. (**C**) Percentage of DEGs in the KO and the KO-T-treated cells. Area of alteration of genes involved in different processes when TKS4 is absent and EZH2 is inhibited. The analysis only included genes that exhibited a minimum two-fold change in expression, either through downregulation or overexpression.

Analysis of the KEGG signaling network revealed that the loss of TKS4 mostly impacts the PI3K/AKT signaling pathway, which aligns with the predicted function and molecular interactions of TKS4 [2]. A major disruption of this cascade can be determined by the greater number of downregulated genes compared to upregulated genes (Figure 6A). However, in response to EZH2 inhibitor treatment, the alterations shifted in the direction of upregulation of genes related to this pathway, indicating the involvement of TKS4 and EZH2 in proper PI3K/AKT signaling. This is also true for the calcium, Ras, Rap1, cAMP, MAPK, AMPK, and Wnt signaling pathways. The genes associated with the JAK/STAT pathway exhibit a relatively balanced upregulation and downregulation of its participant proteins. However, it is also switched to upregulation following EHZ2 inhibitor treatment. Phospholipase D, HIF1, and Fox0 pathways also exhibit similar behavior. Apelin, Hippo, and cGMP pathways are less sensitive to the absence of TKS4, and they did not show alterations upon EZH2 inhibitor treatment either. The genes related to mTOR, NFkB, ErbB, and TNF signaling are mostly upregulated, and the perturbation is reduced upon treatment, suggesting that EZH2 may play a negative regulator role in these pathways in HCT116 cells. Notch, VEGF, TGF-beta, and sphingolipid pathways are little affected by the deletion of TKS4, with a small number of differentially expressed genes, but most of these are upregulated (Figure 6A). Interestingly, while hedgehog and sphingolipid signaling seem to be rather insensitive to the absence of TKS4, they show relatively strong reactions to the inhibition of EZH2 activity (Figure 6A). It is important to highlight that the ultimate outcome of abnormal gene expression cannot be determined solely based on the number of suppressed or elevated genes in a signaling pathway, as the changes in the expression of repressor or activator genes can have contrasting impacts on the overall functioning of the pathway.

As a consequence of the significant alterations in gene expression exhibited in KO cells, various cellular processes are disrupted. Unsurprisingly, signal transduction exhibited the highest number of perturbed genes, with a higher proportion of downregulated examples. EZH2 inhibition appeared to reverse these changes, shifting the balance to the upregulation side. The same could be observed in processes like developmental biology, metabolism, gene expression (transcription), post-translational modification, and receptor tyrosine kinase (RTK) signaling pathways. Remarkably, the TKS4 KO cells exhibited a decrease in the expression of DNA repair and chromatin structure genes, indicating a compromised ability to respond to cellular stress. Cellular senescence, while not among the most affected processes, exhibits more upregulation than downregulation, and no notable alteration is found following the treatment (Figure 6B). Furthermore, our findings indicate that around 65% of widely recognized indicators of senescence exhibit at least 20% alterations in their expression (Supplementary Figure S6), and approximately 15% of them show reactions to EZH2 inhibition (Figure 6C). Splicing and splicing factor markers (Figure 6C, right, and Supplementary Table S1) represent similar behavior upon TKS4 deletion and subsequent EZH2 inhibition. The processes that involve the most genes with altered expression in the TKS4 KO cells are, unsurprisingly, EMT and invasion, and both show strong reactions to EZH2 inhibition (Figure 6C, left).

### 3.7. Changes in Long Non-Coding RNAs

In recent decades, it has been demonstrated that non-coding RNAs (ncRNAs) that do not possess protein-coding capacity play a significant role in regulating different biological processes, a relevance comparable to protein-coding genes. A large group of ncRNAs are long non-coding RNAs (lncRNAs) that have a length above 200 nucleotides [103], containing more than 100,000 representatives in the human genome [104]. Numerous observations underline their involvement in cancer development and progression [105], and several of them are regarded as novel biomarkers for cancer diagnosis and prognosis [106]. 

**Figure 7:**
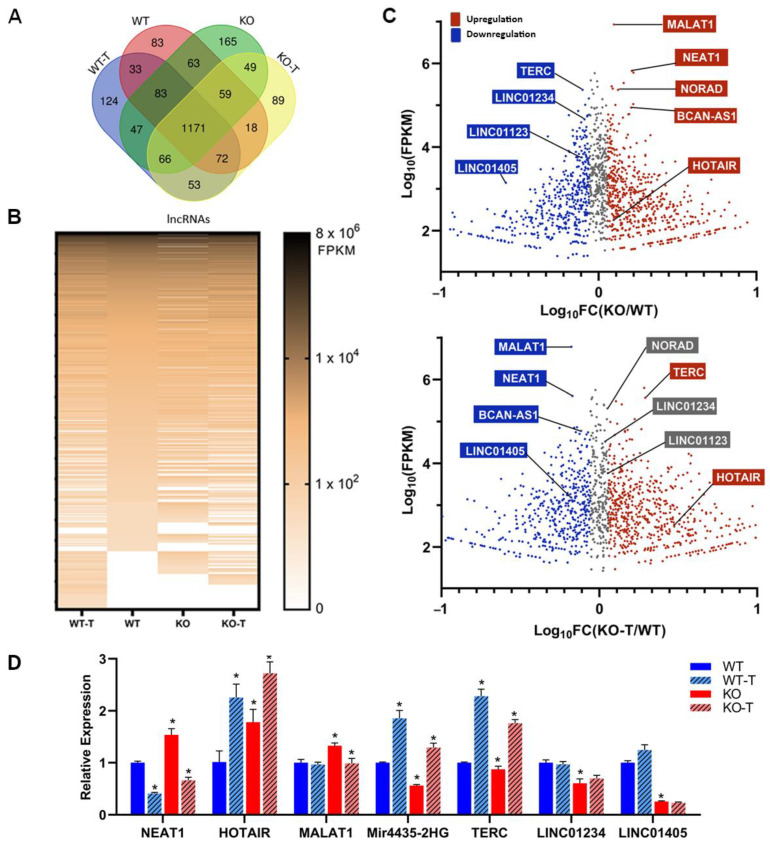
Transcriptomic changes in lncRNAs. (**A**) The Venn diagram and (**B**) heatmap illustrate the changes in non-coding genes generated by TKS4 KO and EZH2 inhibitor-treated cells. (**C**) Several lncRNAs show altered expression levels after TKS4 deletion (top), while EZH2 inhibition restores the expressions in many examples (bottom). (**D**) Quantitative analysis shows fold-changes in the different lncRNAs. Results significant at *p* > 0.005 are marked with an asterisk.

To gain a deeper understanding of the disruptions caused by the TKS4 knockout, we examined the expression of lncRNAs both in WT and TKS4 KO cells. We identified 1870 lncRNAs that were present in our transcriptomic datasets in any of the investigated cell types (Supplementary Table S3). A total of 83 lncRNAs were exclusively identified in WT cells, whereas 165 lncRNAs were only found in KO cells. Additionally, 124 lncRNAs were detected only in WT-T cells, and 89 lncRNAs were only present in KO-T cells (Figure 7A). A heatmap based on the transcriptomic data (Figure 7B) indicates similar large-scale reorganization in the expression of lncRNAs, as was seen in the case of mRNAs (Figure 5B). Interestingly, while TKS4 KO resulted in the overexpression of several mRNAs, this effect was not as pronounced in lncRNAs. The role of EZH2 in the regulation of lncRNA expression is indicated by the several lncRNAs appearing in the EZH2 inhibitor-treated samples (Figure 7B).

However, we discovered that the loss of TKS4 led to the overexpression or downregulation of many lncRNAs that have already been linked to the development of cancer (Supplementary Table S3).

MALAT1 is known for its high expression levels in different cancers, and it was among the most abundant lncRNAs in the WT HCT116 cells (Figure 7C, Supplementary Table S3, and Supplementary Figure S7A), and its expression is further increased in TKS4 KO cells. Its RNA level is decreased upon EZH2 inhibition, just like NEAT1 (both isoforms), NORAD, and BCAN-AS1 (Figure 7C). HOTAIR, a well-known molecular partner of EZH2 that is involved in the regulation of EMT [26,107], was overexpressed in the TKS4 KO cells, and its expression was further increased upon EZH2 inhibition.

The lncRNAs whose expression was downregulated in TKS4 KO cells and were induced upon EZH2 inhibition include Mir4435-2HG, LINC01234, and TERC (Figure 7C). The expression of LINC01123 and LINC01405 was reduced in the TKS4 KO cells and remained unchanged after EZH2 inhibitor treatment, indicating an EZH2-independent regulation. The loss of TKS4 led to a decrease in the expression of lncRNAs, such as LINC02575, HAGLR, LINC00222, and HOTTIP, but they did not react the same way to the EZH2 inhibition. LINC02575 and HAGLR did not show significant changes; the expression of LINC00222 was further decreased, and HOTTIP expression was restored upon treatment. The expression of selected lncRNAs was confirmed with a quantitative qPCR, which resulted in identical results to those provided by the RNA-seq data (Figure 7D, Supplementary Table S3, and Supplementary Figure S7B).

## 4. Discussion

Our results, based on transcriptomic sequencing and quantitative PCR, clearly indicate that TKS4 KO cells display diverse expression levels of several conventional EMT markers, and these variations, at least partially, are mediated by the histone methyltransferase EZH2. We observed a marked increase in the activity of EZH2 in the TKS4 KO cells, which manifested in a marked increase in the H3K27me3 mark levels. As this histone modification results in gene expression inhibition, this indicates that the genes that show significant downregulation in the KO cells that can be reversed by EZH2 inhibitor treatment are most probably under the direct control of the enzyme. However, if EZH2 controls the expression of a different transcriptomic inhibitor, its overactivity can also manifest in elevated gene expression in an indirect manner. Nevertheless, a connection can only be assumed if the EZH2 inhibitor treatment is capable of reversing the effects of TKS4 removal.

We could see examples of both of these scenarios in our system, as proteins specific to epithelial cell types were reduced in expression, but most of them could recover after inhibitor treatment. Meanwhile, mesenchymal markers showed increased expression. However, Twist1 and N-cadherin are downregulated, and no alteration was found in the EMT main marker, SNAI1. These alterations suggest a partial EMT (pEMT) and align with previous findings involving the TKS4 KO cell line [9]. We propose that the hyperactivity of EZH2 is responsible for the induced p-EMT because most of the phenotypic changes could be reversed by EZH2 inhibitor treatment. EZH2 and EMT are intricately linked, as stated in the literature [108,109,110]. The important role of EZH2 in our system is also indicated by the fact that MMPs, which are significant promoters of EMT and invasion and are mostly upregulated in TKS4 KO cells, are predominantly restored upon EZH2 inhibitor treatment.

In addition to the observed pEMT, the observed increase in invasion capacity of the TKS4 KO cells is also mediated by EZH2, as most of the invasion markers were upregulated in the TKS4 KO cells but could be reduced by EZH2 inhibition. This observation is mostly in correlation with the literature [111,112,113], in which EZH2 is shown to enhance tumor cell invasion.

In line with the known physiological role of TKS4 [1,7], its removal led to a widespread decrease in the expression of genes associated with the formation of invadopodia and podosomes [114,115]. However, we could also detect upregulation of invadopodia and podosome-related genes that could be reversed by EZH2 activity inhibition.

**Figure 8:**
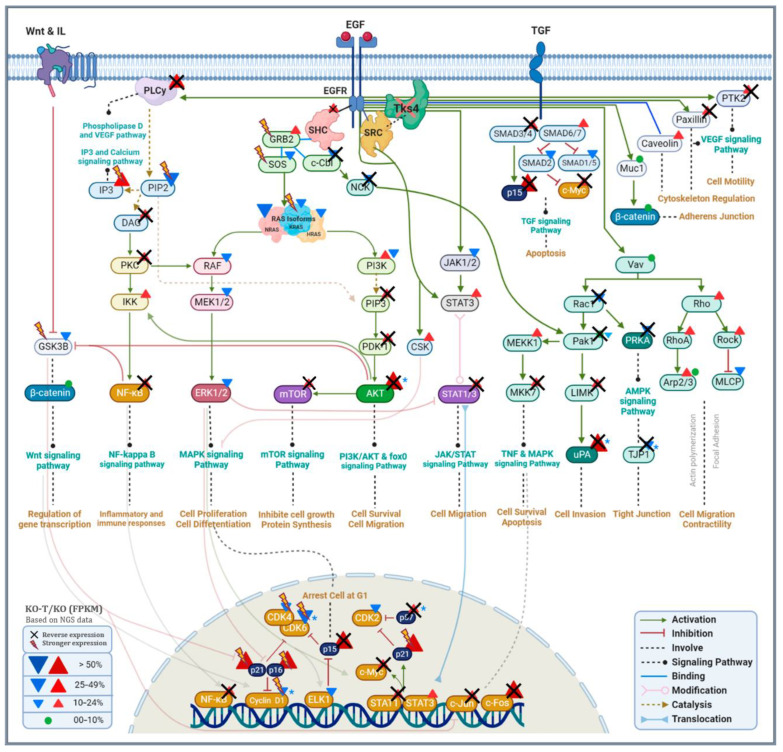
Schematic representation of the signaling pathways affected by the deletion of Tks4 and EZH2 inhibition. TKS4 deletion triggers alterations across several signaling pathways. EZH2 inhibition reversed the expression of many genes, indicating the involvement of EZH2 in these signaling pathways. Gene expression changes are illustrated by triangles, with red indicating upregulation and blue indicating downregulation. If both are present, they indicate alterations in distinct subunits. The cross indicates decreased expression with EZH2 inhibition, while lightning shows increased expression with EZH2 inhibition. Core genes within a certain pathway are shaded with darker colors. The diagram compares KO samples with WT samples, whereas the treatment with EZH2 inhibitors (cross and lightning) is based on comparing KO-T samples with KO samples. Gene expression changes validated by RT-qPCR are indicated by blue asterisks. The figure was adapted with appropriate modifications from our previous publication [35].

TKS4 KO cells experience a notable decrease in their proliferation rate, despite the fact that in most cases, proliferation increases in parallel with migration, EMT, and invasion [116]. However, the morphological observations and the gene expression alterations all provided evidence of the decrease in proliferation. The TKS4 KO cells exhibit downregulation of multiple genes in the MAPK cascade (mainly responsible for cell proliferation), which are either not affected by EZH2 inhibition or become even more repressed after treatment (Figure 8). This indicates that the decreased proliferation was due to the disruption caused by TKS4 deletion and not by EZH2 hyperactivity.

Removal of TKS4 leads to a major dysregulation of several signaling pathways through global alterations in gene expression, indicating the existence of intracellular factors that are involved in spreading and amplifying the effect of its absence. Based on our results detailed above, EZH2 and, through its activity, PRC2 appear to be one of these factors. A closer analysis of the signaling pathways reveals the possible molecular mechanisms of EZH2 involvement in the translation of TKS4 loss into gene expression changes. We have provided a schematic representation of the signaling pathways that are affected by the lack of TKS4, and we found that EZH2 inhibition reversed the expression of many genes, indicating the involvement of EZH2 in different signaling pathways (Figure 8).

We detected an increased expression of multiple genes associated with the PI3K/Akt pathway, indicating that EZH2 suppresses their negative regulator(s). One of the upregulated genes, PLAU is known to induce cancer cell migration and invasion [117] while decreasing cell proliferation [118], which was observed in the cells lacking TKS4 (Figure 8). The AKT3 overexpression demonstrates increased activity of this pathway, and elevated AKT3 expression is correlated with the process of EMT in colorectal cancer cells [119]. AKT3 expression decreased after treatment, indicating that controlling EZH2 activity might moderate colorectal cancer progression. 

EZH2 has been implicated in PI3K/AKT signaling by several earlier studies [120,121,122,123], which is confirmed by our observations as well. The hyperactivity of EZH2 in the TKS4 KO cells may be a consequence of cell cycle-related kinase (CCRK or CDK20), as was observed in human hepatocarcinogenesis. CCRK induces EZH2 upregulation and phosphorylation in an epigenetic circuit. We detected a slight elevation in the expression of CCRK in the TKS4 KO cells (Supplementary Table S2), but it was insensitive to EZH2 inhibition, making it unlikely that this circuit is a major contributor to the observed effects in our system.

JAK/STAT is another pathway that promotes cell migration, proliferation, and differentiation [124] and is majorly affected by TKS4 deletion. It is also connected to EZH2, as several of the gene expression alterations could be reversed by EZH2 activity inhibition (Figure 6 and Figure 8). This connection is also confirmed by literature data, as EZH2 has been shown to interact with STAT3 [125], and activation of JAK/STAT3 leads to an increase in the phosphorylation of STAT3 and EZH2 mediated by phosphorylated AKT [126], leading to the activation of EZH2.

Based on our observations, the genes involved in other cancer-related pathways, such as NFκB [127,128,129], VEGF [130,131,132,133], and mTOR [134,135,136], are all at least partially regulated by EZH2 in HCT116 cells in connection with TKS4 (Figure 8).

An important aspect of our study is the effect of TKS4 deletion on the expression of different lncRNAs and their connection to EZH2. Gaps in the functional annotation of lncRNAs complicate the in-depth analysis of our results in terms of possible molecular mechanisms, but a considerable number of the identified lncRNAs have already been shown to interact with EZH2 and participate in the regulation of the affected signaling pathways. MALAT1 is known to bind to EZH2 and direct it to specific chromatin regions, leading to tumor suppressor gene repression [137,138,139], it can activate the EZH2-Notch1 and the PI3K/Akt signaling pathways. The overexpression of MALAT1 in the TKS4 KO cells could be reversed by EZH2 inhibition, suggesting that EZH2 may be a negative regulator of this lncRNA. Importantly, MALAT1 expression was not sensitive to EZH2 inhibition in the WT cells, indicating that EZH2 involvement in the regulation of MALAT1 expression may be related to the pathways perturbed by TKS4 deletion. Since apparently MALAT1 binding to EZH2 can inhibit tumor suppressor gene expression, overactivation of EZH2 in the KO cells may lead to a positive feedback loop, accelerating the malignant processes. HOTAIR is another well-known lncRNA partner of EZH2, as it was suggested to regulate cell cycle progression through EZH2 [140], and it induces cell invasion and metastasis through the PRC2 complex [107,141]. Similar to MALAT1, HOTAIR was also upregulated in the TKS4 KO cells, but its expression was further increased upon EZH2 inhibition both in WT and KO cells. This observation suggests a complex, indirect involvement of EZH2 in the regulation of HOTAIR transcription. NEAT1 promotes migration, invasion, metastasis, and EMT via interacting with EZH2 as a scaffold RNA [142,143,144], but it can also interact directly with EZH2 and recruit PRC2 to specific gene promoters [145,146,147]. NEAT1 has been found to mainly modulate the Wnt/β-catenin signaling pathway [144] and is sensitive to EZH2 inhibition in the WT and KO cells alike, supporting the hypothesis that EZH2 regulates an inhibitor of NEAT1 expression.

Another lncRNA, MIR4435-2HG, is probably under the transcriptional control of EZH2, as its downregulation in the KO cells could be reversed by EZH2 inhibition. Reduction in MIR4435-2HG levels led to decreased proliferation rates in other studies [148,149,150], indicating its possible regulatory role, but in our system, the elevation of MIR4435-2HG could not restore the proliferation rate in KO cells.

## 5. Conclusions

The above-detailed results unequivocally indicate that TKS4 deletion results in the overactivation of EZH2, which is realized in large-scale transcriptomic changes. Our analysis extended over the protein-coding transcripts, and we could identify several non-coding RNAs that are affected as well. As most of the observed phenotypic changes in cell migration, EMT, invasion, and cell proliferation could be reversed or mitigated by the inhibition of EZH2 activity, we can conclude that EZH2 is directly or indirectly involved in the signaling pathways connected to TKS4. EZH2 involvement could occur as a result of the redistribution of intracellular signaling molecules, like CCAR1, which is a supposed interactor of TKS4 [10], or through the dysregulated expression of EZH2-interacting molecules, like lncRNAs. As TKS4 functions as a scaffold and plays a role in the phosphorylation cascade, it might also be necessary to study the downstream phosphorylation cascade to comprehend the exact nature of this connection. However, additional research is necessary to elucidate the precise connection between TKS4 and EZH2.

## Data Availability

The datasets generated and analyzed during the current study are available from the corresponding author on request.

## Supplementary Materials

The following supporting information can be downloaded at: https://www.mdpi.com/article/10.3390/biom14040445/s1, Figure S1: (a) Western blotting picture of TKS4, Ezh2, H3K27me3, and mono-methyl lysine; (b) ChIP-seq data analysis; (c) Western blotting picture of H3K27me3 testing two different concentrations of DZNep and viability of cells compared to different concentrations of DZNep; Figure S2: (a) Migration assay picture; (b) EMT markers based on RNA-seq data; Figure S3: (a) Invasion assay picture; (b) invasion markers based on RNA-seq data; Figure S4: Proliferation markers based on RNA-seq data; Figure S5: Quantitative analysis of mRNA genes confirming RNA-seq data; Figure S6: Senescence promoting and inhibiting markers based on RNA-seq data; Figure S7: Gene expression level of different lncRNAs compared to GAPDH control and different lncRNAs relative expression based on transcriptomic analysis in different conditions of cells compared to WT; Figure S8: ICC picture shows the localization changes in MMP14 in TKS4 KO cells compared to WT cells; Table S1: List of genes related to specific cellular processes based on log2FC, KEGG, and Reactome. Table S2: List of the differentially expressed mRNAs; Table S3: List of deferentially expressed lncRNAs; Table S4: List of antibodies and probes used in the current study.
