# Supplementary material for: Morphological Changes Induced by TKS4 Deficiency Can Be Reversed by EZH2 Inhibition in Colorectal Carcinoma Cells
Source: Biomolecules. 2024 Apr 5;14(4):445. doi: 10.3390/biom14040445 (PMC11047920; doi:10.3390/biom14040445)
Supplement: Supplementary file 1 [file biomolecules-14-00445-s001.zip › Supplementary Figures.pdf]

A

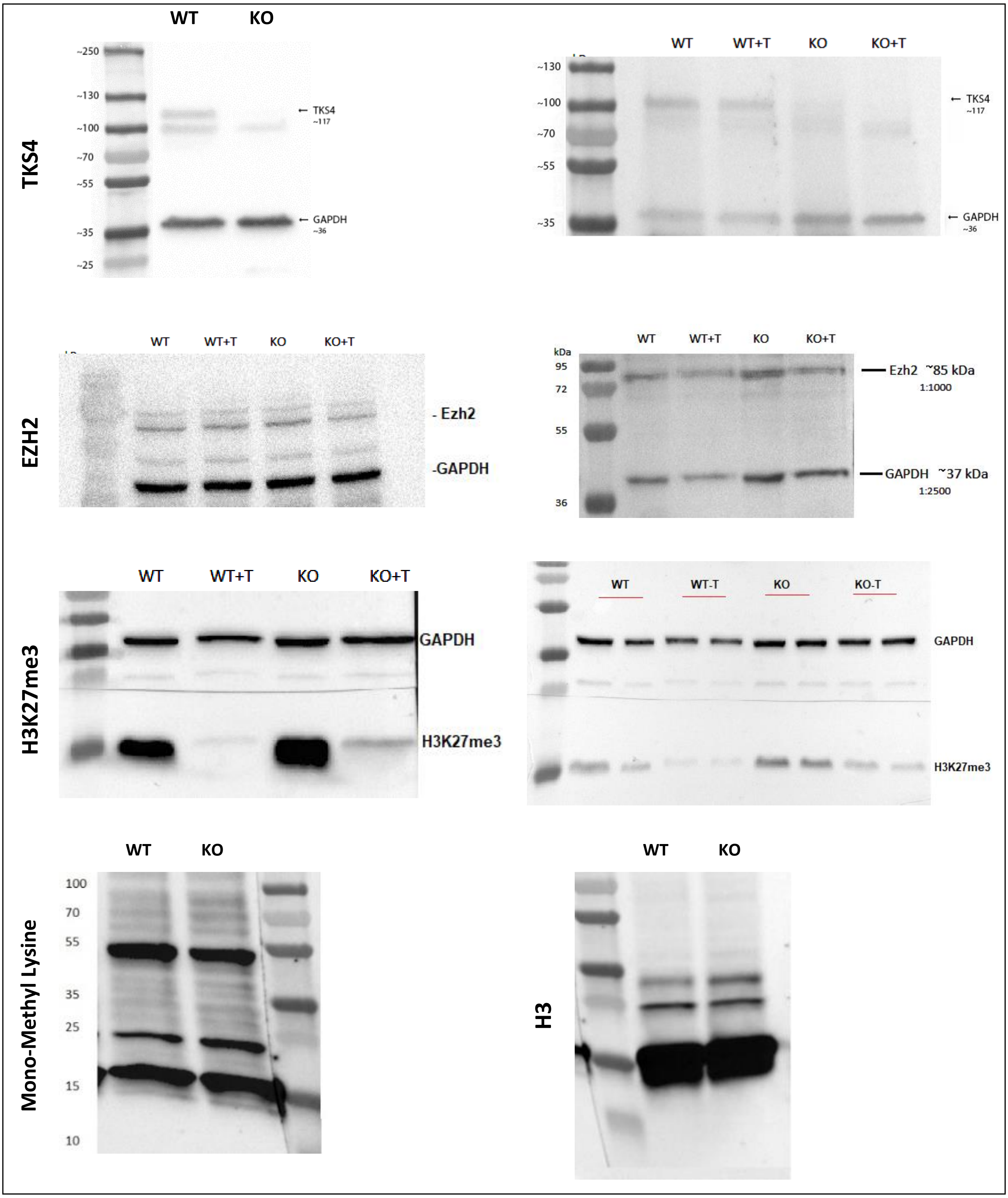

TKS4
WT
KO
~250
~130
~100
~70
~55
~35
~25
← TKS4
~117
← GAPDH
~36
WT
WT+T
KO
KO+T
~130
~100
~70
~55
~35
← TKS4
~117
← GAPDH
~36
EZH2
WT
WT+T
KO
KO+T
- Ezh2
-GAPDH
kDa
95
72
55
36
Ezh2 ~85 kDa
1:1000
GAPDH ~37 kDa
1:2500
H3K27me3
WT
WT+T
KO
KO+T
GAPDH
H3K27me3
WT
WT-T
KO
KO-T
GAPDH
H3K27me3
Mono-Methyl Lysine
WT
KO
100
70
55
35
25
15
10
H3
WT
KO

B

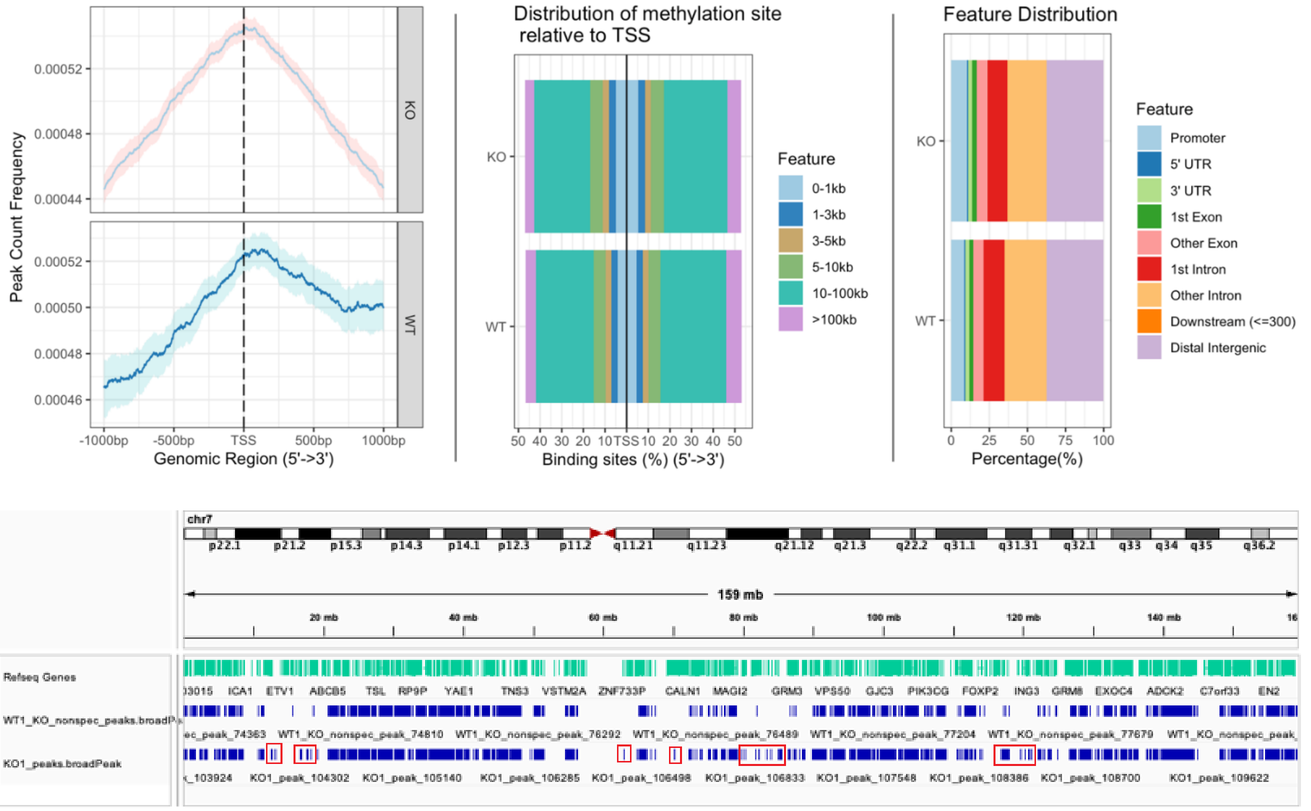

C

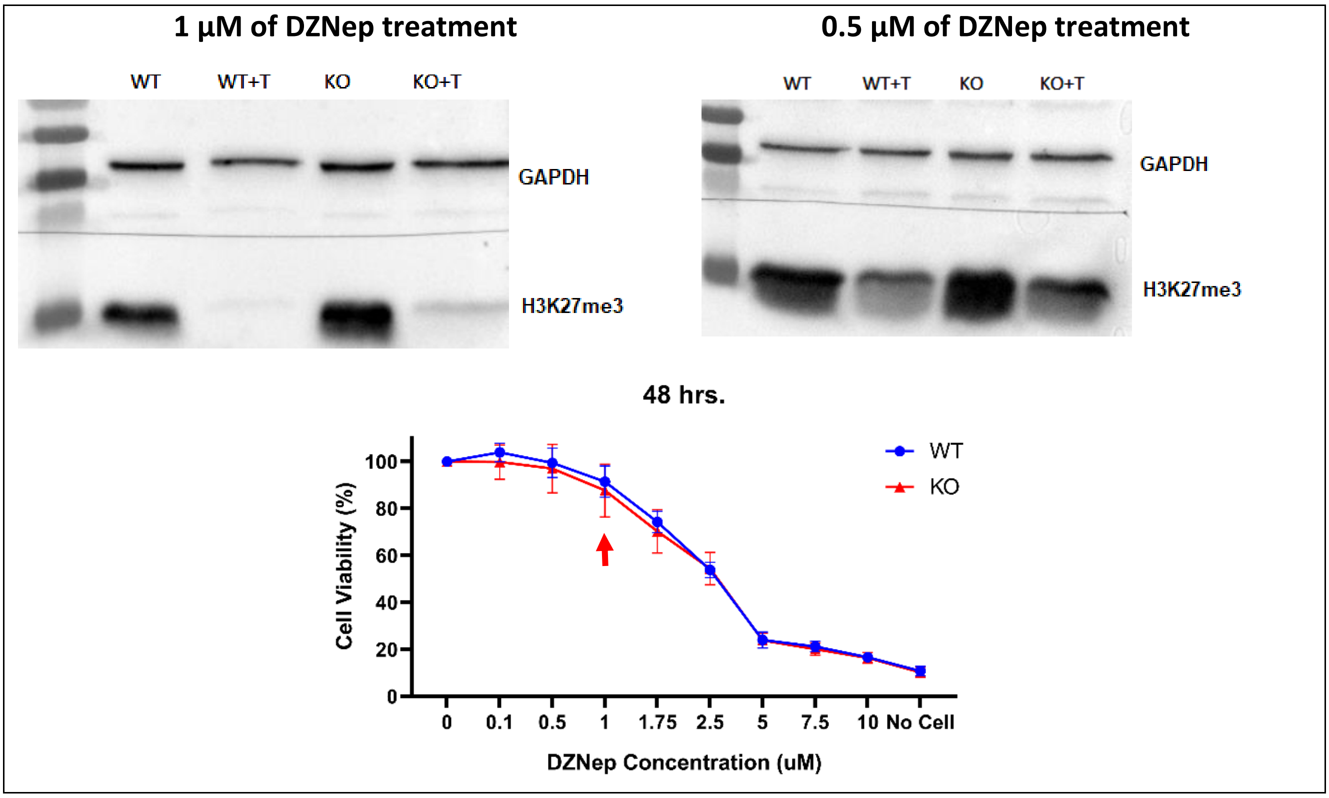

Figure S1. (**a**) Western blotting images of TKS4, Ezh2, H3K27me3 and Mono-Methyl Lysine. (**b**) ChIP-seq data analysis. Top panel indicates the increased number of peaks in KO cells compared to WT, especially in the promoter regions. Lower: Red rectangles indicate peaks only present in the KO samples. (**c**) Top: Western blotting picture of H3K27me3 testing two different concentrations of DZNep. Bottom: Viability of cells in response to different concentration of DZNep used.

A

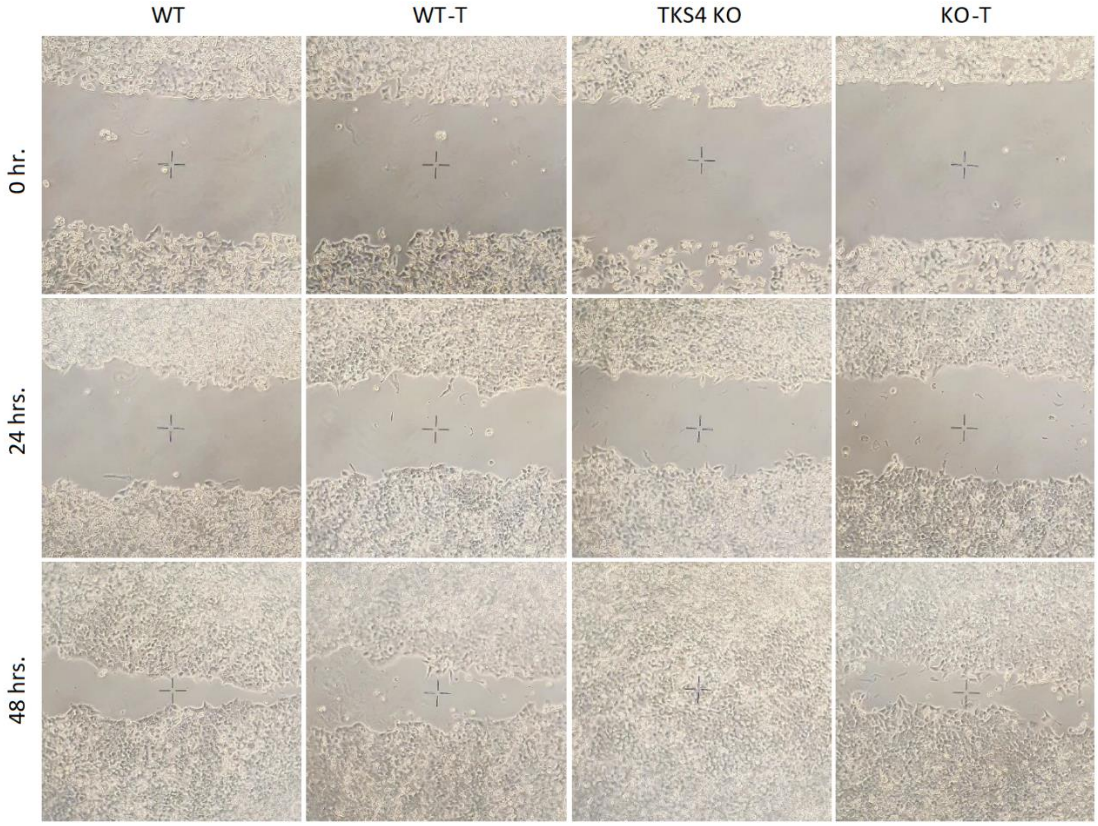

B

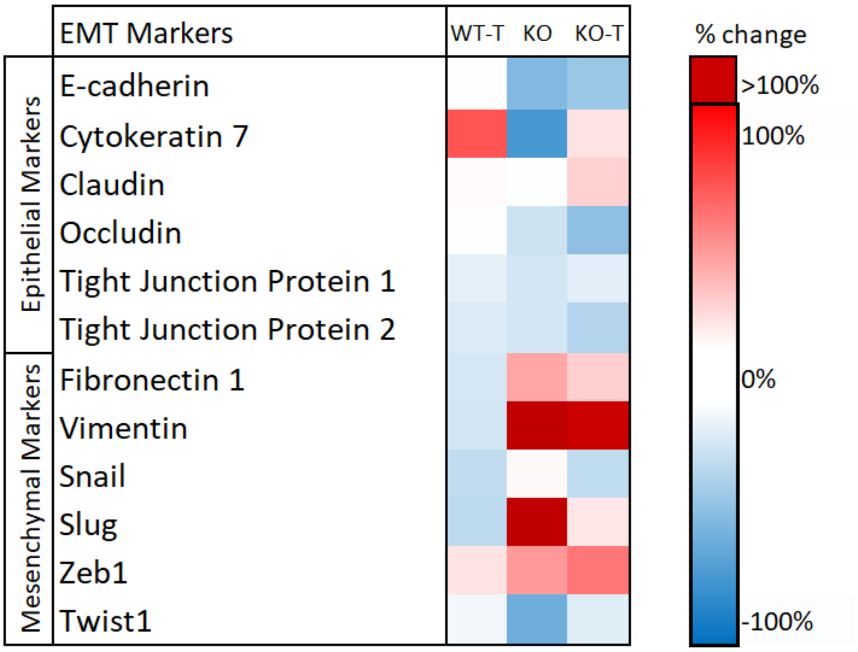

Figure S2. (**a**) Migration assay images at 0, 24, and 48 hours. Images of the untreated samples are adapted from our previous publication [35]. (**b**) Expression of EMT markers based on RNA-seq data in different conditions of cells compared to WT.

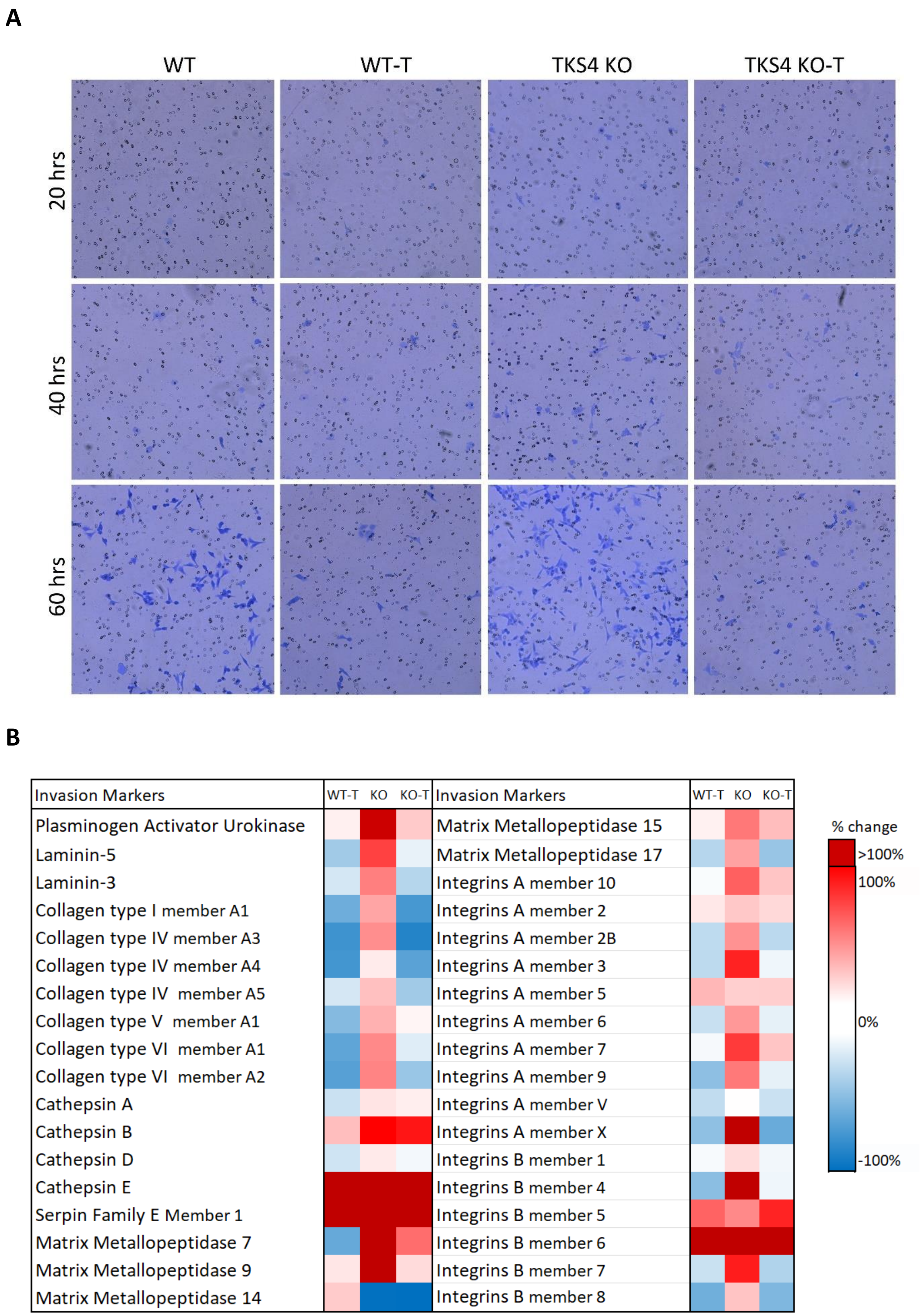

Figure S3. (**a**) Invasion Assay images at 20, 40, and 60 hours. Images of the untreated samples are adapted from our previous publication [35]. (**b**) Expression levels of invasion markers based on RNA-seq data in different conditions of cells compared to WT.

Proliferation Markers	WT-T	KO	KO-T	Proliferation Markers	WT-T	KO	KO-T
Marker Of Proliferation Ki-67				Cyclin Dependent Kinase 2			
Cyclin A2				Cyclin Dependent Kinase 4			
Cyclin B1				Cyclin Dependent Kinase 6			
Cyclin B2				Cyclin Dependent Kinase 7			
Cyclin B3				Cyclin Dependent Kinase 8			
Cyclin C				Coiled-Coil Domain Containing 25			
Cyclin D1				Cell Division Cycle 25C			
Cyclin E1				MCM2 (Minichromosome Maintenance Complex)			
Cyclin E2				MCM3			
Cyclin F				MCM4			
Cyclin H				MCM5			
Cyclin I Family Member 2				MCM6			
Cyclin J				MCM7			
Cyclin K				MCM8			
Cyclin Q				MCM9			
Cyclin Dependent Kinase 1				MCM10			

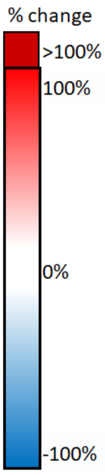

Figure S4. Expression of proliferation markers based on RNA-seq data in different condition of cells compared to WT.

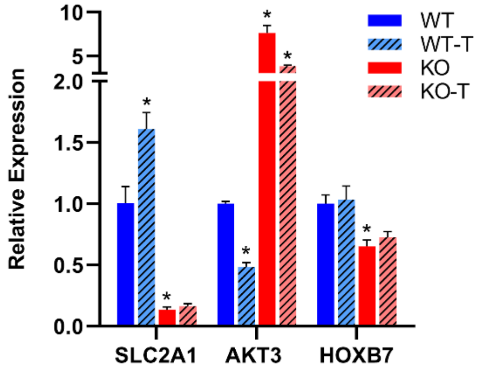

Figure S5. Quantitative analysis of selected mRNAs to validate RNA-seq data

Senescence Promoting Markers	WT-T	KO	KO-T	Senescence Inhibiting Markers	WT-T	KO	KO-T
Tumor Protein P53				Pyruvate Dehydrogenase E1 Subunit Alpha 1			
p16				Cyclin Dependent Kinase 1			
p15				Cyclin Dependent Kinase 2			
p21				Cyclin Dependent Kinase 4			
p27				Cyclin Dependent Kinase 6			
p57				Cyclin D1			
p19ARF				Cyclin E1			
p38MAPK				Cell Division Cycle 25C			
PDP2 (Pyruvate Dehydrogenase Phosphatase)				MCM2 (Minichromosome Maintenance Complex)			

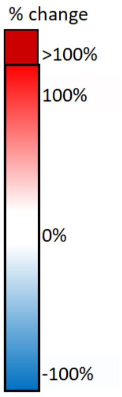

Figure S6. Senescence promoting and inhibiting markers based on RNA-seq data in different conditions of cells compared to WT.

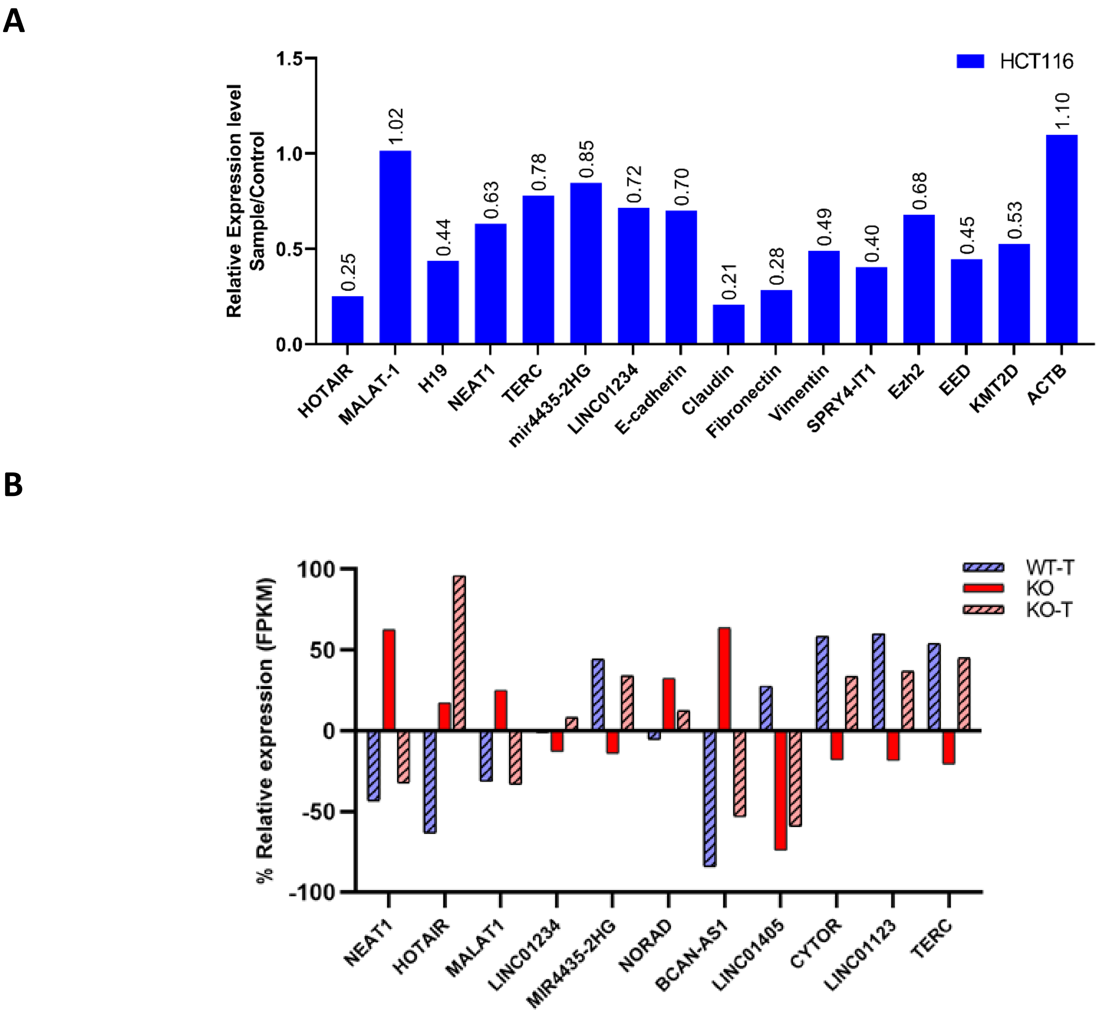

Figure S7. (**a**) Expression levels of different lncRNAs compared to GAPDH control based on the Ct (Cq) of qPCR. (**b**) Relative expression levels of selected LncRNAs based on transcriptomic analysis in different conditions of cells compared to WT (WT=0 line).

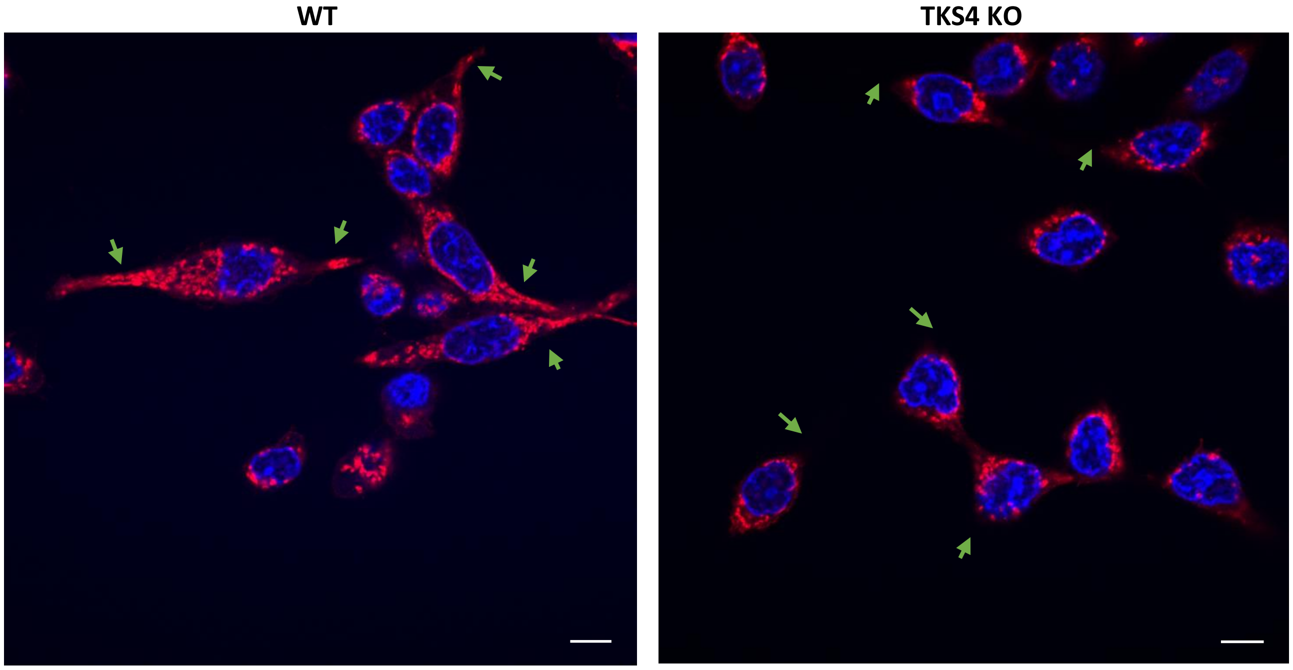

Figure S8. ICC images show the localization changes of MMP14 in TKS4 KO cells compared to WT cells. Blue: DAPI, red: MMP14. Differences in MMP14 localization indicated by green arrows. Scale bar: 20 µm.
